# Mutation Analysis of *BRCA1, BRCA2, PALB2* and *BRD7* in a Hospital-Based Series of German Patients with Triple-Negative Breast Cancer

**DOI:** 10.1371/journal.pone.0047993

**Published:** 2012-10-24

**Authors:** Franziska Pern, Natalia Bogdanova, Peter Schürmann, Min Lin, Aysun Ay, Florian Länger, Peter Hillemanns, Hans Christiansen, Tjoung-Won Park-Simon, Thilo Dörk

**Affiliations:** 1 Clinics of Obstetrics and Gynaecology, Hannover Medical School, Hannover, Germany; 2 Clinics of Radiation Oncology, Hannover Medical School, Hannover, Germany; 3 Fluidigm Corporation, San Francisco, California, United States of America; 4 Institute of Pathology, Hannover Medical School, Hannover, Germany; IFOM, Fondazione Istituto FIRC di Oncologia Molecolare, Italy

## Abstract

Triple-negative breast cancer (TNBC) is an aggressive form of breast carcinoma with a poor prognosis. Recent evidence suggests that some patients with TNBC harbour germ-line mutations in DNA repair genes which may render their tumours susceptible to novel therapies such as treatment with PARP inhibitors. In the present study, we have investigated a hospital-based series of 40 German patients with TNBC for the presence of germ-line mutations in *BRCA1*, *BRCA2*, *PALB2*, and *BRD7* genes. Microfluidic array PCR and next-generation sequencing was used for *BRCA1* and *BRCA2* analysis while conventional high-resolution melting and Sanger sequencing was applied to study the coding regions of *PALB2* and *BRD7*, respectively. Truncating mutations in *BRCA1* were found in six patients, and truncating mutations in *BRCA2* and *PALB2* were detected in one patient each, whereas no truncating mutation was identified in *BRD7*. One patient was a double heterozygote for the *PALB2* mutation, c.758insT, and a *BRCA1* mutation, c.927delA. Our results confirm in a hospital-based setting that a substantial proportion of German TNBC patients (17.5%) harbour germ-line mutations in genes involved in homology-directed DNA repair, with a preponderance of *BRCA1* mutations. Triple-negative breast cancer should be considered as an additional criterion for future genetic counselling and diagnostic sequencing.

## Introduction

Triple-negative breast cancers (TNBCs) account for about 15% of all invasive breast cancers and are defined as tumors that lack expression of estrogen receptor (ER) and progesterone receptor (PR), and do not show overexpression of HER2/neu [1]. TNBCs are usually high-grade, invasive ductal carcinomas and have been linked with a worse prognosis [1], [2]. There is some overlap with a basal-like pattern of gene expression [2], [3]. Women with a breast cancer family history experience a significantly increased risk of triple-negative breast cancer [4]. Importantly, carriers of mutations in the breast cancer susceptibility gene 1, *BRCA1*, frequently have basal-like and/or triple-negative breast cancers [5].

Triple-negative cancers which harbour a dysfunctional *BRCA1* pathway may be sensitive to platinum-based chemotherapy [6] and to inhibitors of the poly(ADP-ribosyl)-polymerase that selectively target cells deficient in homologous recombination DNA repair [7], [8]. Recent studies have indicated that germ-line *BRCA1* mutations might be overrepresented in patients with TNBC, in particular those with an early onset of the disease [9]–[11]. Deleterious mutations of the second breast cancer susceptibility gene, *BRCA2*, have also been reported to occur at a high frequency in German patients with triple-negative breast cancers [12].

The BRCA1 protein is involved in homologous recombinational DNA repair through an interaction with BRCA2 that is mediated by PALB2, the partner and localizer of BRCA2 [13], [14]. PALB2 bridges the BRCA1 and BRCA2 proteins and regulates their function in the DNA damage response [14]. *PALB2* mutations have been associated with breast cancer in several studies [15]–[19]. The BRCA1 protein is also involved in the transcriptional regulation of the estrogen receptor alpha (ESR1) through an interaction with bromodomain-containing protein 7 (BRD7), a subunit of the SWI/SNF chromatin remodelling complex. BRD7 is required for the recruitment of BRCA1 to the *ESR1* promoter [20]. BRD7 also interacts with the tumour suppressor p53 and is required for efficient transcription of a subset of p53 target genes [21], [22]. *BRD7* is frequently deleted in human breast tumours harbouring wildtype p53 [21] but the potential role of *BRD7* germ-line mutations in breast cancer has not yet been fully elucidated.

In the present study, we scanned the whole coding regions of *BRCA1*, *BRCA2*, *PALB2* and *BRD7* in order to investigate the relative contributions of germ-line mutations in these genes to triple-negative breast cancer in a hospital-based series of German patients.

## Patients and Methods

### Patients

For the present study we ascertained 40 patients who were diagnosed with triple-negative breast cancer during the years 2009–2011 at the Clinics of Obstetrics and Gynaecology at Hannover Medical School. Medical records were reviewed with the following information captured on a case report form: demographics (age, date of birth, ethnicity), personal history of cancer, age of diagnosis, recurrence, current status, family history from the time of the patient’s diagnosis. Median age at onset was 52 years (range 22–81 years). 35 of the 40 patients were of German descent, the others were Polish, Tunesian, Korean, Iranian or Filipino. 12 of the 40 patients reported a first-degree family history of breast cancers, two of them also with a first-degree family history of ovarian cancer. The expression of ERα, PR and HER2/neu was assessed using mAB SP1 (ERα), mAB 1E2 (PR), mAB 4B5 (Her2), mABs XM26 and LL002 (CK5/14), and mAB 2-1E1(EGFR). Antigen was retrieved by automatically pouring retrieval solution (Ventana) onto sections with subsequent heat treatment. After quenching of endogenous peroxidase activity by immersion in 3% H_2_O_2_ for 10 min, tissue sections were incubated with primary antibody at room temperature followed by staining using the UltraView kit (Ventana). All cases showing less than 1% tumor cells expressing ERα or PR and all cases showing an HER2 score of less than 2 were considered negative. For CK5/14 and EGFR a semiquantitative score (0 no expression; 1 weak expression; 2 moderate expression and 3 strong expression) as well as the relative percentage of positive tumor cells was calculated.

Informed written consent was obtained from each patient, and the study was approved by the Institutional Review Board at Hannover Medical School (Ethics commission vote No. 762/10). For each patient, genomic DNA was isolated from peripheral white blood cells using standard phenol-chloroform extraction.

### BRCA1 and BRCA2 Analysis

Target-specific primers were designed by Fluidigm Corp (San Francisco) using Fluidigm primer service program with the following recommendations: Tm range of 59–61°C, max of homopolymer is 3 and GC% less than 65%. Common sequence tags (CS1 and CS2) were added to forward and reverse primers for Access Array amplicon tagging experiments. 77 primer pairs were designed and validated to cover all exons except exon 22 (74 bp) in *BRCA1* (Figure S1). The exons of *BRCA1* and *BRCA2* were then amplified from triple-negative breast cancer patients to receive 40 pools of 77 amplicons. For this purpose, each genomic DNA sample was normalised to a concentration of ∼50 ng/ml and loaded onto an Access Array (Fluidigm, San Francisco), a microfluidic array in which a PCR was performed with nested primer pairs. Each primary primer pair contained the template-specific sequence and a tag sequence. Each secondary primer pair with sample contained the anti-tag sequence, a sample-specific unique barcode, and the 454 adaptor sequence. PCR products were harvested from each sample and were checked on an agarose gel to confirm uniformity of the amplicon coverage (distribution within 2-fold). PCR products were subsequently pooled using 1 µl per sample and purified, the library was subjected to emulsion PCR and the products were pyrosequenced with a GS FLX 454 system (Roche, Basel).

Sequencing data were analysed with NextGENe 2nd Generation Sequencing Software v.2.2.1 (SoftGenetics, Philadelphia, U.S.A.). In brief, raw data were converted to FASTA files and were aligned to *BRCA1* and *BRCA2* gbk files from the human reference sequences (GRCh37.p5 Primary Assembly, NC_000017.10 for *BRCA1* and NC_000013.10 for *BRCA2*; http://www.ncbi.nlm.nih.gov/genbank/). Only reads over 25 bases were converted, and reads were rejected if they contained more than 3 uncalled bases. Alignment was performed with a required matching of over 85% within more than 50 bases. This yielded an average of 2.35 million converted reads per sample (range 1.05–3.43×10^7^), and about 95% of the reads could be matched. The average read length per sample was 487 (range 481–491) bases, and the average coverage per sample was 74-fold (range 33–110 fold). The average coverage per exon was above 30-fold (30–240 fold) except for three amplicons (exons 5, 15, and 21 of *BRCA1*) that were covered less than 20-fold; these three exons and the missing exon 22 of *BRCA1* were manually resequenced using BigDye Terminator Cycle sequencing (Applied Biosystems) with exon-flanking intronic primer pairs. For the others, mutation filters were set to exclude mutations with a percentage less than 10% or less than 3 counts, and to exclude homopolymer indels with a forward/reverse balance less than 0.1. Two regions were further inspected manually in each sample as they were largely represented by only one sequenced strand. All identified mutations, apart for common polymorphisms or known synonymous variants, were finally validated by conventional Sanger sequencing using BigDye chemistry and a 3100 Avant Genetic Analyser (Applied Biosystems, Darmstadt).

### PALB2 Analysis

All exons of *PALB2* were scanned for mutations by high-resolution melting (HRM) analysis as previously described [17]. In brief, PCR amplifications were set up in the presence of the EvaGreen dye (BioBudget, Krefeld, Germany), and high-resolution melting analysis was performed on the Rotor-Gene 6000 real-time PCR machine (Corbett Research, Mortlake, Australia). Melting profiles were evaluated using the Melt Curve Analysis tool of the Rotor-Gene 6000 Series Software Version 1.7. All samples with suspicious melting behaviour were then subjected to direct sequencing to identify the underlying substitution using BigDye chemistry and a 3100 Avant Genetic Analyser (Applied Biosystems, Darmstadt).

### BRD7 Analysis

To study genetic variants in the *BRD7* coding region, DNA samples were analysed by conventional Sanger sequencing. Primer pairs were designed to amplify each of the 17 coding exons (exons 2–18 of *BRD7*) including their flanking intron sequences. Primer sequences and PCR conditions are given in Table S1. All PCR products were sequenced using BigDye terminator chemistry v1.1 on a 3100 Avant Genetic Analyser (Applied Biosystems, Darmstadt); the call rate was 100%.

### mRNA Analyses

LCLs were established by EBV immortalisation of peripheral white blood cells [23] and were cultured in RPMI 1640 supplemented with 15% heat-inactivated FCS and 1 mM L-glutamine (Biochrom, Berlin, Germany) at 37°C under an atmosphere of 5% CO_2_. Total mRNA was extracted from cell pellets using a modified guanidinium isothiocyanate/phenol protocol, and 1 µg RNA was reversely transcribed with a First Strand cDNA synthesis kit following the manufactureŕs instructions (GE Healthcare). One-fifth of the cDNA was included into a PCR with primers 5′-CTG CAA AGA AGC TGT TGC AC-3′(c7F) and 5′-CTT CCA GTT GTC ATT CCC AG-3′ (c11R) spanning the exons 7 through 11 of the *BRD7* transcript. RT-PCR products were separated by 2% agarose gel electrophoresis, stained with GelRed and evaluated on a UV transilluminator.

### Statistics and Bioinformatics

Genotype frequencies were compared using chi-square analyses, and p<0.05 was considered significant. Fisheŕs exact test was used for numbers <5. Sequence variants were checked for previously published reports in the BIC database (http://research.nhgri.nih.gov/projects/bic/Member/index.shtml), in the *PALB2* mutation database (http://www.lovd.nl/PALB2), in the NCBI SNP database (http://www.ncbi.nlm.nih.gov/snp), or in the PubMed database (http://www.ncbi.nlm.nih.gov/pubmed). Exonic sequence variants in *BRD7* were tested for possible effects on binding sites of splicing factors SF2/ASF, SC35, SRp40 or SRp55 using ESE Finder 3.0 (http://rulai.cshl.edu/tools/ESE).

## Results

**Figure 1:**
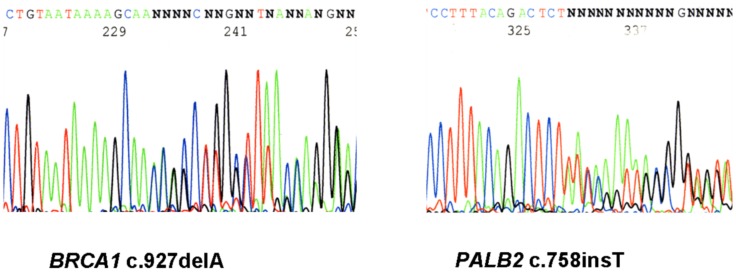
Double heterozygosity for *BRCA1* and *PALB2* mutations. A case with digenic mutations in *BRCA1* (left) and *PALB2* (right). Left: Heterozygosity for mutation c.927delA in exon 10 of the *BRCA1* gene. Right: Heterozygosity for mutation c.758insT in exon 4 of the *PALB2* gene. The sense strand is shown in both electropherograms.

We investigated a hospital-based series of 40 consecutive patients with triple-negative breast cancer for mutations in the whole coding sequences of *BRCA1* and *BRCA2* as well as in the two genes *PALB2* and *BRD7*, which encode interaction partners of the BRCA1 protein. Next-generation sequencing of barcoded *BRCA1* and *BRCA2* amplicon pools revealed five truncating mutations of *BRCA1* in six patients (6/40, 15%) and one truncating mutation of *BRCA2* in one patient (1/40, 2.5%) (**Table 1**). All six mutations create premature stop codons upstream of the penultimate exon and were considered pathogenic. Three of the *BRCA1* mutations were known breast cancer- associated mutations that were recorded in the Breast Cancer Information Core (BIC) database. Two of the remaining three frame-shift mutations, c.843_846del4 in *BRCA1* and c.5238insT in *BRCA2,* were not included in the BIC database but had recently been reported to the NCBI SNP database as rs80357792 and rs80359500, respectively. We did not find a reference for *BRCA1* mutation c.927delA indicating that this could be a private mutation (**Figure 1**).

**Table 1:** Truncating mutations in *BRCA1*, *BRCA2* and *PALB2* among 40 TNBC patients.

Gene	Mutation	Exon	Consequence	Pathology	BIC*
*BRCA1*	c.843_846del4	10	frameshift	Bilateral, medullary/undiff., AD 43 and 53 ys,1 first-deg OC, 1 second-deg BC.	no
	c.927delA	10	frameshift	Multifocal ductal, AD 65 ys, 1 first- deg BC(AD 48 ys) and 2 second- deg BC (AD 35–40 ys).IHC: EGFR pos., CK5/14 neg.	no
	c.4689C>G	15	p.Y1563X	Ductal, AD 49 ys, 1 second-deg BC (AD 63 ys),1 first-deg OC (AD 47 ys). IHC: CK5/14 pos.	yes
	c.5153-2delA	IVS18	exon skipping	Ductal, AD 62 ys, 1 first-deg BC. IHC: EGFR neg.,CK5/14 weakly pos.	yes
	c.5266dupC	20	frameshift	Ductal, AD 38 ys, death at 1 year after diagnosis.1 second-deg BC (AD 30 ys),; IHC: CK5/14 pos.	yes
	c.5266dupC	20	frameshift	Ductal, AD 51 ys, 1 first-deg BC and 9 second-degBC/OC. IHC: CK5/14 neg.	yes
*BRCA2*	c.5238insT	11	frameshift	Ductal, AD 62 ys, 1 second-deg BC. IHC: EGFRweakly pos., CK5/14 weakly pos.	no
*PALB2*	c.758insT	4	frameshift	Multifocal ductal, AD 65 ys, 1 first- deg BC(AD 48 ys) and 2 second- deg BC (AD 35–40 ys).IHC: EGFR pos., CK5/14 neg.	n.a.

Truncating mutations in *BRCA1*, *BRCA2* and *PALB2* among 40 TNBC patients. Mutations were designated according to the improved mutation nomenclature recommended by the Human Genome Variation Society (www.hgvs.org/mutnomen/). AD = age at diagnosis, BC = breast cancer, OC = ovarian cancer, IHC = immunohistochemistry, n.a. = not applicable.

* BIC database as from Sep 29, 2010 (http://research.nhgri.nih.gov/projects/bic/Member/index.shtml), accessed on July 10, 2012.

Some clinical features of the TNBC patients with *BRCA1* or *BRCA2* mutations are summarized in Table 1. Two of the seven carriers had bilateral breast cancer. Three of the seven carriers had been diagnosed below age 50, compared with 15 out of 33 non-carriers (p = 0.9). Five of the seven carriers had a first-degree family history of either breast or ovarian cancer, compared with nine out of 33 non-carriers (p = 0.03). Six of the seven carriers showed ductal histology, while one *BRCA1* mutation carrier had a medullary carcinoma. All seven carriers were of German descent though the carrier of the *BRCA1* c.843_846del4 mutation had a Polish background. Mutation carriers were still alive at 1–3 years following standard taxane-based chemotherapy, except for one *BRCA1* c.5266dupC carrier who died at one year after diagnosis.

We furthermore scanned the whole coding region of *PALB2* in all TNBC patients using high-resolution melting analysis [17]. Apart from known polymorphisms, one sample was identified with an aberrant melting profile and was subsequently sequenced in exon 4 of the *PALB2* gene. The patient turned out to be heterozygous for a novel truncating mutation, c.758insT (Figure 1). The insertion of a nucleotide into exon 4 of *PALB2* results in a frame-shift and creates a premature termination signal three codons downstream, within the same exon (p.S236X). This *PALB2* mutation was identified in the same patient who also carried the novel *BRCA1* mutation, c.927delA, and therefore is a double heterozygote for mutations in both genes. The patient was diagnosed with a multifocal invasive ductal carcinoma of stage pT2 and grade 3. Lymph nodes were positive (pN2a, 4/13), but she had no metastases. She also presented with large myomas of the uterus, a small meningioma, visual disturbances due to chorioid neovascularisation, and bipolar disorder. She underwent mastectomy and received chemotherapy with epirubicin and cyclophosphamide which was well tolerated, and she has shown no signs of recurrence three years after diagnosis. One of her three sisters had died from breast cancer by the age of 48 years, and two of three daughters of this sister also had premenopausal breast cancer. No further cancer was known in this family.

We finally considered *BRD7* as a plausible candidate gene for TNBC, based on the known interaction of its gene product with BRCA1 in the regulation of estrogen receptor expression. Primer pairs were designed to amplify all 17 coding exons and the flanking intron sequences of *BRD7*, and the TNBC samples were investigated by conventional Sanger sequencing. We identified two alterations in the coding region both of which were synonymous: The known polymorphism c.846C→T in exon 8 (rs1062348) was found at a minor allele frequency of 0.18, which was similar to its reported frequency of 0.24 in the SNP database, and the novel variant c.1861C→T (TCC→TCT; p.S618S) in exon 17 was identified in a single patient. Bioinformatic assessment using ESE Finder 3.0 did not predict alterations in splicing for the rare c.1861C→T variant whereas a new binding site for the splicing factor SC35 was predicted for the minor allele of the c.846C→T polymorphism. However, subsequent inspection of mRNA from two lymphoblastoid cell lines representing both homozygous genotypes did not reveal any alternative splicing in either of both lines (data not shown), indicating that c.846C→T does not disturb *BRD7* splicing, at least in lymphoid cells. Altogether, this study did not reveal pathogenic *BRD7* alterations and thus the role of germ-line mutations in *BRD7* for TNBC, if any, is much less pronounced than the role of *BRCA1* mutations in our series of German patients (6/40, p = 0.03).

## Discussion

Triple-negative breast cancers (TNBCs) have received much interest at the clinical, biological and epidemiological level due to the aggressive behaviour of the tumour, the poor prognosis and the present inapplicability of therapies with receptor antagonists. Among new therapies that are under investigation [24], the most important one is PARP inhibition, which induces synthetic lethality in tumour cells deficient in homology-directed DNA double-strand break repair such as cells mutated in *BRCA1* or *BRCA2* [7], [8]. *BRCA1* mutated tumours are often triple-negative, and the treatment with PARP inhibitors might be promising in at least some TNBC patients [25]. Several recent studies on TNBC have focussed on *BRCA1* mutations whereas the prevalence of mutations in *BRCA2* or mutations in other genes of the BRCA1 pathway has been less extensively studied. This prompted us to perform a full scanning of the coding region of *BRCA1*, *BRCA2*, *PALB2* and *BRD7* in a consecutive series of German TNBC patients who have visited our hospital over a two-years-period.

*BRCA1* and *BRCA2* exons were analysed using a Fluidigm Access Array followed by 454 sequencing to save costs and to achieve a high throughput. Combining a microfluidic amplification system with massive parallel sequencing is an effective method for mutation scanning, which has previously been employed for the diagnostics of familial hypercholesterolemia [26] and here was applied to *BRCA1* and *BRCA2* sequencing. This approach identified a total of seven heterozygotes for truncating mutations in *BRCA1* or *BRCA2* among the 40 TNBC patients in our series (17.5%) which were all confirmed by Sanger sequencing. These included two patients with *BRCA1* mutation c.5266dupC that is common in Eastern Europe and Germany [27]. The rate of 17.5% may be an underestimate as our approach would not have identified large genomic deletions or far intronic mutations. There was no association with earlier age at diagnosis, however more mutation carriers than non-carriers had a first-degree family history of breast or ovarian cancer. Despite this trend towards a positive family history, two of the seven patients would not have been eligible for mutational screening under current guidelines [12] though one of the two would have been detected with expanded criteria including TNBC patients younger than 50 years [28].

*BRCA2* mutations were underrepresented in our study compared with *BRCA1* mutations which seems to be in contrast with a recent report from another German series of TNBC patients who were also unselected for age at diagnosis and family history [12]. The latter study identified five mutations in *BRCA2* and one mutation in *BRCA1* in 30 German TNBC patients, whereas we have identified six mutation carriers for *BRCA1* and one for *BRCA2* in 40 German TNBC patients. Our results are in line with published results from other study populations that consistently find a higher prevalence of mutations in *BRCA1* than in *BRCA2* [29]–[33]. Furthermore, *BRCA1* mutations were strongly associated with hormone-receptor negative status in previous analyses of high-risk breast-ovarian cancer families whereas most BRCA2-mutated tumours do not have a TNBC phenotype [34]–[38]. The biological reason for the different outcomes remains unclear, given that BRCA1 and BRCA2 collaborate in the homology-directed DNA repair pathway, but BRCA1 may exert a particular role in hormone receptor expression. It has also been discussed that *BRCA2* mutation carriers may develop TNBC later in life than *BRCA1* carriers, and the relatively old age at diagnosis in our *BRCA2* mutation carrier may be in line with this hypothesis [33]. Genetic variation at other loci, such as *BABAM1* (*MERIT40*) on chromosome 19q13.1, might further influence the outcome towards a triple-negative phenotype [39].

Similar considerations may apply to *PALB2*, encoding the partner and localiser of BRCA2 [13]. Mutations in *PALB2* are quite rare, and *PALB2* was mutated in only one TNBC patient in our series. The *PALB2* mutation, c.758insT, was identified in a patient who also carried a *BRCA1* mutation, c.927delA, and thus may provide the first example of a case with digenic mutations in these both genes. Double heterozygotes have previously been reported for *BRCA1* and *BRCA2* mutations and, in one study, appeared to have an earlier onset and a more severe disease than their female relatives carrying a single *BRCA1* or *BRCA2* mutation [40], [41]. While the *BRCA1/PALB2* double heterozygous patient described here had no early onset, the multifocal occurrence of the disease and her positive family history may be consistent with a severe predisposition. Given the *BRCA1* mutated background, it cannot be decided which of the genes drives the triple-negative phenotype in this patient. Most previous studies have not found evidence that *PALB2* mutations were associated with hormone-receptor negative breast tumours [16]–[18], [42], but one study reported that carriers of a Finnish founder mutation in *PALB2* had triple negative breast cancer significantly more often than other breast cancer patients [43]. Although the absence of additional carriers in our series argues against an important role of *PALB2* mutations in German TNBC patients, a possible link between *PALB2* and a triple-negative phenotype still warrants further investigation.

The *BRD7* gene was included as an additional candidate gene for TNBC because BRD7 is required for the BRCA1-mediated transcriptional regulation of the estrogen receptor [20]. *BRD7* has been described as a tumour suppressor gene that is frequently deleted in human breast tumours harbouring wildtype p53 [21] but no clearly pathogenic *BRD7* germ-line mutations have been identified in German patients with familial breast cancer, thus far [44]. In our study, there was no pathogenic mutation detected in any of the 40 TNBC patients, excluding a prominent role of *BRD7* in the genetic predisposition to this cancer. As our sequencing approach was based on Sanger methodology for *BRD7*, the absence of mutations was not due to low coverage of certain regions. Although it remains possible that large genomic deletions or far-intronic mutations may exist which would have escaped our detection, there is presently no evidence to implicate *BRD7* mutations in the etiology of TNBC.

In summary, this study confirms in a hospital-based setting a substantial proportion of high-penetrance germ-line mutations in German patients with triple-negative breast cancer, with a preponderance of *BRCA1* mutations over mutations in *BRCA2* or *PALB2*, and with the exemplification of double heterozygosity for mutations in *BRCA1* and *PALB2*. Triple-negative breast cancer should be considered as an additional criterion for future genetic counselling and diagnostic sequencing.

## Supporting Information

Figure S1 **Primers for** ***BRCA1*** **and** ***BRCA2.*** Barcode primers within *BRCA1* and *BRCA2*, comprised with the adapter sequences for 454, a 10-bp barcode sequences and the common sequence tags.
(TIF)

Table S1 **Primers for** ***BRD7*** **.** Primers sequences for PCR amplification of the coding exons of *BRD7* are shown in 5′->3′ direction. Annealing temperatures ranged between 60–66°C.
(DOC)
