# Supplementary figures and images for: Mutation Analysis of BRCA1, BRCA2, PALB2 and BRD7 in a Hospital-Based Series of German Patients with Triple-Negative Breast Cancer
Source: PLoS One. 2012 Oct 24;7(10):e47993. doi: 10.1371/journal.pone.0047993 (PMC3480465; doi:10.1371/journal.pone.0047993)

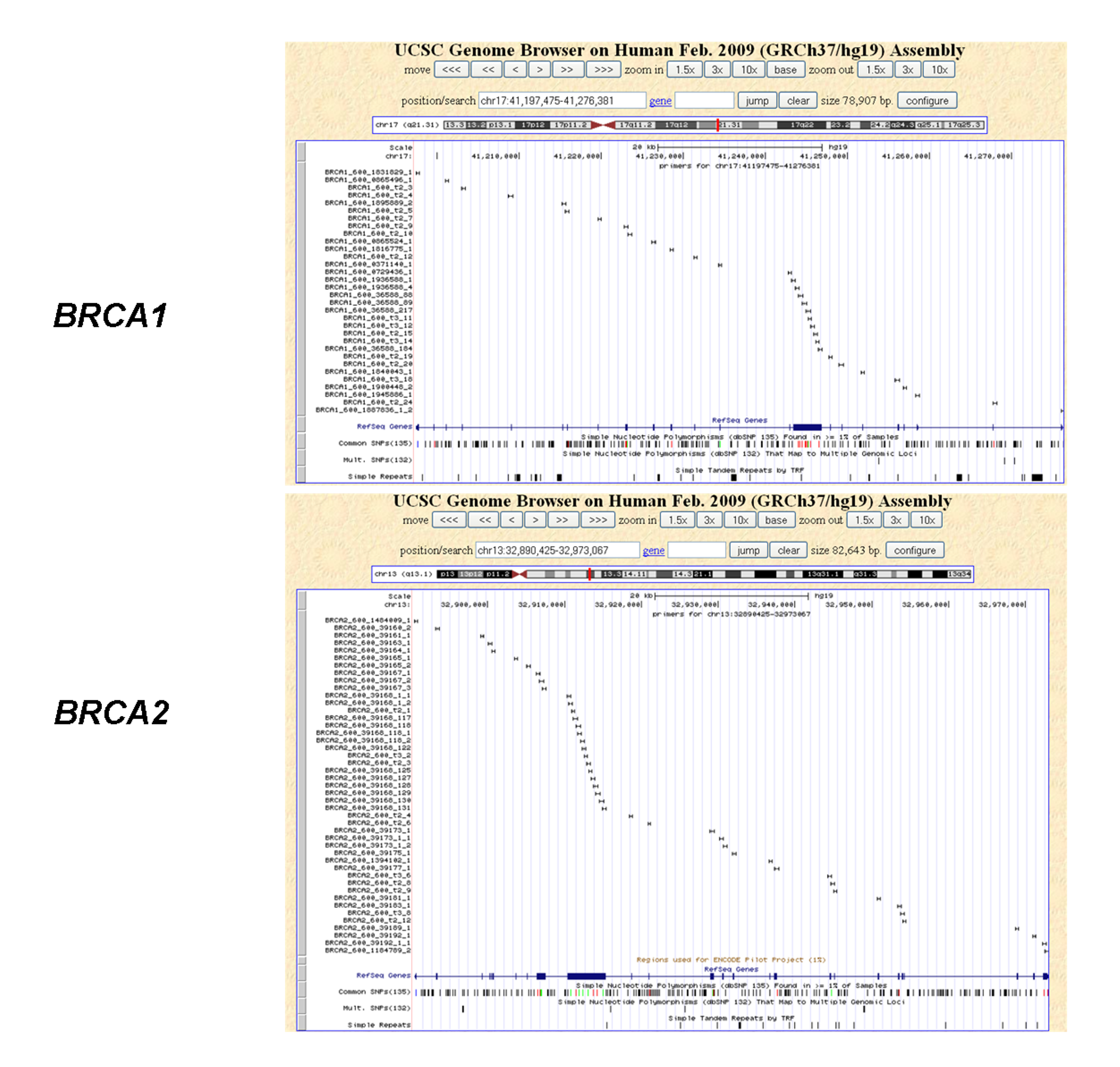

Supplement: Figure S1 — Primers for BRCA1 and BRCA2. Barcode primers within BRCA1 and BRCA2, comprised with the adapter sequences for 454, a 10-bp barcode sequences and the common sequence tags. (TIF) [file pone.0047993.s001.tif]
