# Supplementary material for: Mutation Analysis of BRCA1, BRCA2, PALB2 and BRD7 in a Hospital-Based Series of German Patients with Triple-Negative Breast Cancer
Source: PLoS One. 2012 Oct 24;7(10):e47993. doi: 10.1371/journal.pone.0047993 (PMC3480465; doi:10.1371/journal.pone.0047993)
Supplement: Table S1 — Primers for BRD7 . Primers sequences for PCR amplification of the coding exons of BRD7 are shown in 5′->3′ direction. Annealing temperatures ranged between 60–66°C. (DOC) [file pone.0047993.s002.doc]

**Table S1**: Primers used for *BRD7* sequencing

| **Exon** | **Forward Primer** | **Reverse Primer** |
| --- | --- | --- |
| 2 | GACCGGAAGCGGCGCCGCA | GGAAAGAGCGGAAGCCCGGCA |
| 3 | AGCCCCGATTTTAATCGACG | AAAATGAGGTTGGCACCTGG |
| 4 | GGCAGTAAAGTACATTGAGC | CAAAGCTCCAACTTAGAAGC |
| 5 | GCTTCTAAGTTGGAGCTTTG | TGGAGTACTTCTGCTGCCAG |
| 6 | TTCTGCTCCTAGACCAATAG | TCTGGTTTTCCATCACCAAG |
| 7 | TTTTCAACCTGGGGGTGCAG | TTACTATGGCCAACAAACAG |
| 8 | CTGGTTAATGACAGGATAAG | CCTTACTAGCTTGAAGAAGAC |
| 9 | CAGGTCATCAAGGAAGTAAG | TTTCAGATGCCCCAAATCAG |
| 10 | CTCTGGTATCAAGCATTTCGG | GTGCATAATGACACAGACTG |
| 11 | ATGCAGTGGTTTGTGCGGAG | AGATATGGTGCCCACCTAG |
| 12 | GATTTGCAAACCAGCCAATG | ACTGCAGATGCTACTATCAG |
| 13 | GGATAAAGCCAGTGGAGGTG | TTCAGGGCCACAGAGTTCAG |
| 14 | CCTTGATATTCGCCTTCATG | GGCCTTCCCATTCTCAAGAC |
| 15 | TCTAGTTAACACTGCTGTGG | CTTGACTGCCTACATCATGC |
| 16 | GATTCGGTTTACTGCACAAG | CTGTCCCTGGGATTCACATC |
| 17 | GGGATGCCTCTATCTAAAGG | ATACCAAATCCGAGGTCCTG |
| 18 | AGCAGTTATGCTTCTGGATG | GAATGCTGCACAGGTATGGC |
